# Glycogen synthase kinase GSK3α promotes tumorigenesis by activating HIF1/VEGFA signaling pathway in NSCLC tumor

**DOI:** 10.1186/s12964-022-00825-3

**Published:** 2022-03-15

**Authors:** Xiaonian Cao, Wei Wu, Dao Wang, Wei Sun, Senyan Lai

**Affiliations:** 1 Department of Thoracic Surgery, Tongji Hospital, Tongji Medical College, Huazhong University of Science and Technology, Wuhan, 430030 China; 2 Department of Gastrointestinal Surgery Center, Tongji Hospital, Tongji Medical College, Huazhong University of Science and Technology, Wuhan, 430030 China; 3 Department of Hepatopancreatobiliary Surgery, The Central Hospital of Wuhan, Tongji Medical College, Huazhong University of Science and Technology, Gusao Tree Road No. 16 of Jianghan District, Wuhan, 430000 Hubei Province China

**Keywords:** GSK3α, HIF1α, Tumor angiogenesis

## Abstract

**Background:**

Lung cancer is one of the most common cancers and the leading cause of cancer-related death. Glycogen synthase kinase-3 (GSK-3) α, a member of the glycogen synthase kinase-3 family, reportedly plays a role in tumorigenesis. However, its biological function in tumorigenesis requires deeper exploration. Hypoxia is a major feature of solid tumor, along with decreasing availability of oxygen, inducing treatment resistance, and tumor progress.

**Methods:**

Levels of GSK3α expression in clinical samples were detected using western blot and IHC assays, while its biological function and underlying mechanism of action in tumor progression were investigated using western blot, CCK8, cell cycle, colony formation, Transwell, ELISA and tube formation assays. Furthermore, we investigated the relationship between GSK3α expression and the HIF1α/VEGFA signaling pathway in vivo using a mouse xenograft model.

**Results:**

GSK3α was significantly upregulated in NSCLC patients with cases that exhibited high GSK3α levels recording shorter survival times. Moreover, GSK3α overexpression promoted proliferation, migration, invasion and clone formation ability of NSCLC cells, while its silencing resulted in an opposite phenomenon. Moreover, GSK3α not only activated the HIF1α/VEGFA signaling pathway, but also regulated HIF1α stabilization independently via the PHDs-pVHL signaling pathway. Moreover, GSK3α-mediated tumor angiogenesis depended on HIF1α expression both in vitro and in vivo.

**Conclusion:**

GSK3α functioned as an oncogene in NSCLC tumorigenesis by regulating the HIF1/VEGFA signaling pathway in an independent manner through the PHDs-pVHL signaling pathway. These findings were expected to provide novel sights to guide future development of therapies for effective treatment of NSCLC.

**Video abstract**

**Supplementary Information:**

The online version contains supplementary material available at 10.1186/s12964-022-00825-3.

## Background

Lung cancer is one of the most common types of cancers and the leading cause of cancer-related deaths worldwide [1, 2]. Previous studies have shown that almost 1.8 million people are diagnosed with lung cancer, while 1.6 million cases die of the disease every year [3]. The 5-year survival rate of patients with lung cancer is about 4–17%, depending on stage and regional differences [4, 5]. Notably, approximately 85% of all new lung cancer cases comprise the non-small-cell lung cancer (NSCLC) subtype [3, 6]. Although anti-angiogenesis treatment has been proven effective for treatment of the disease, the therapy is accompanied by drug-resistance [7–9]. Therefore, further explorations into the molecular mechanism underpinning NSCLC progression is imperative to identification of targets to guide development of effective treatment therapies.

Glycogen synthase kinase-3 (GSK-3) α is a member of the glycogen synthase kinase-3 family [10]. That differs from GSK3β at its N- or C-terminal sequences. Previous studies have shown that loss of GSK3β induced death in development in mice, with animals lacking GSK3α found to exhibit abnormalities in glucose metabolism and in the brain. Most researches have focused on the function of other members of the GSK3 family in tumorigenesis, with only a handful of reports describing the role of GSK3α in this process [11, 12]. For example, GSK3α was found to play a more important role than GSK3β in survival of cancer cells, during resistance to bortezomib-induced cancer treatment [10]. These results showed that both genetic factors play different biological functions in tumorigenesis, although that of GSK3α in the process remains largely unknown.

Hypoxia is a major feature of solid tumors, and is further characterized by a decrease in availability of oxygen, induction of resistance to treatment, and tumor progression [9, 13]. Notably, hypoxia not only contributes to tumor plasticity and heterogeneity, but also promotes tumor aggressiveness and metastatic ability [14]. A hallmark of this progression is upregulation of hypoxia-inducible factor 1α expression, coupled with abnormal elevation of vessel angiogenesis [15, 16]. Oxygen concentration has been shown to regulate HIF1α stability, and its expression level. In normoxia, HIF1α is subject to hydroxylation by HIF prolyl hydroxylase domain family proteins (PHDs), while the von Hippel–Lindau tumor suppressor protein (pVHL) can bind to hydroxylated HIF1α, thereby promoting recruitment of an E3 ubiquitin ligase complex and subsequently initiating proteasomal degradation of HIF-α [17–19]. However, HIF1α cannot be hydroxylated under hypoxia, although it can be independently regulated via the PHDs-pVHL signaling pathway. For example, previous studies have shown that p53 protein is not only stabilized under hypoxia, but further promotes HIF1α degradation via the ubiquitin–proteasome pathway [13, 20]. Moreover, anti-angiogenesis has been shown to be an effective treatment strategy for tumor patients [21]. Although it is commonly accompanied by drug-resistance [22]. Recent studies have shown a positive correlation between anti-angiogenesis resistance and upregulation of HIF1α expression in patients [23–25].

In the study, we found that GSK3α was significantly upregulated in NSCLC patients, who subsequently exhibited shorter survival times. Moreover, GSK3α overexpression promoted proliferation, migration, invasion and clone formation ability of NSCLC cells, while knocking out this gene inhibited the aforementioned phenomena. Elucidation of the underlying mechanism of action revealed that GSK3α not only regulates the HIF1α/VEGFA signaling pathway, but also modulates HIF1αstabilization independently via the PHDs-pVHL signaling pathway. Moreover, GSK3α-mediated tumor angiogenesis relies on HIF1α expression both in vitro and in vivo. Collectively, these findings reveal a novel mechanism of NSCLC progression and are expected to guide development of therapies for NSCLC treatment.

## Materials and methods

### Cell lines and antibodies

A549, H226, HUVEC and HEK 293 T cells were purchased from the American Type Culture Collection (ATCC, Manassas, VA, USA). HUVECs and A549 cells were cultured with F-12 K medium. H226 cells were cultured with 1640 medium. HEK 293 T cells were cultured with DMEM medium. All mediums were added fetal bovine serum to a final concentration of 10%. All cells were cultured with 5% (v/v) CO2 at 37 °C. For hypoxia, cells were cultured with 1% (v/v) O2 for 6 h. Antibodies for GSK3α (4337; 9338), β-actin (3700), HIF1β (5537; 3414), HIF1α-OH (3434), and HIF1α (36,169; 79,233) were purchased Cell Signaling Technology. Antibodies for VEGFA (ab52917), and flag (ab205606) were purchased from Abcam company.

### Clinic samples

Clinic samples and matched normal samples were obtained from NSCLS patients at Tongji Hospital, Tongji Medical College Huazhong University of Science and Technology. This study was approved by the Huazhong University of Science and Technology Ethics Committee.

### Quantitative real time PCR

mRNAs of cells were isolated using the TRIzol (Invitrogen, Carlsbad, CA, USA), and then were performed according to the manufacturer’s instructions. Quantitative PCR was conducted using an ABI 7300 real-time PCR system (Applied Biosystems). The primers of RT-PCR were listed in Additional file 1: Table S1.

### Cell viability assay

The CCK8 cell viability kits were bought from ABclonal Technology (RM02823; ABclonal). Cell viability assays were conducted according to the instruction book.

### Cell cycle analysis

Pre-treated cells were washed with cold phosphate-buffered saline (PBS) three times and fixed with 80% ethanol overnight at − 20 °C. Next, cells were washed PBS twice and then stained with PI for 10 min at room temperature. Cell cycle distribution was measured by the Becton–Dickinson FACScan System (Franklin Lakes, NJ, USA).

### Clone formation assays

100–400 pre-treated cells were seeded into 6-well plates. The medium was replaced every 3 days for 2 weeks. Next, cells were fixed with 4% formaldehyde for 30 min and then stained with 0.1% crystal violet. Colonies’ images were gained using a microscope.

### Transwell assays

Pre-treated 1 × 10^4^ (for migration) or 1 × 10^4^ (for migration) cells with serum-free medium were seeded into the upper chamber. 500ul medium plus 10% FBS was added into the lower chamber. Mitomycin C (10 μg/ml) was used to inhibit cell proliferation. After 16 h, cells were fixed with 4% formaldehyde for 30 min and then stained with 0.1% crystal violet. mitomycin C (10 μg/ml).

zCells on the upper membrane were removed using swabs. Images for cells were gained using a microscope.

### Western blot and immunoprecipitation (IP) analysis

Cells were collected, washed with cold PBS for three times, and the lysed with NP-40 lysis buffer at 4 °C for 30 min. Protein concentration was measured using bicinchoninic acid assay kit (Thermo). Separate protein extracts by electrophoresis in a premade sodium dodecyl sulfate–polyacrylamide minigel (Tris–HCL SDS-PAGE), and then transferred toto PVDF membranes. The membranes were incubated with primary antibodies at 4 °C overnight and then incubated with secondary antibodies. The signaling was detected using a chemiluminescence method. For immunoprecipitation, primary antibodies were incubated with Protein L Magnetic Beads (HY-K0205; MCE) at room temperature for 2 h, and then whole cell lysis was cultured with beads at 4 °C overnight, followed by western blot analysis.

### Tube formation assay

96-Well plates were coated with 100 μl Matrigel. HUVECs (1 × 10^4^) were seeded into each well and cultured with conditional mediums for 12 h. Images was gained using a microscope.

### Enzyme-linked immunosorbent assay (ELISA)

ELISA kits (RK00023) were purchased from ABclonal technology. ELISAs were conducted according to the instruction book.

### Immunohistochemistry (IHC)

Immunohistochemistry was conducted using methods reported previously.

### Mouse xenograft studies

The mouse xenograft experiment was approved by the Animal Care and Use Committee of Tongji Hospital. Four-week-old male BALB/c mice were purchased from Beijing Huafukang Bioscience Company. 2 × 10^6^ pre-treated cells were injected subcutaneously into the back. Tumor volume was measured using 0.5 × Length (L) × Width (W)^2^.

### Statistical analysis

All data were analyzed using SPSS 20.0 software. Differences were measured using the Student’s t test for two groups and by ANOVA for multiple groups. *P* < 0.05 was statistically significant.

## Results

### GSK3α was upregulated in NSCLC tissues

**Fig. 1:**
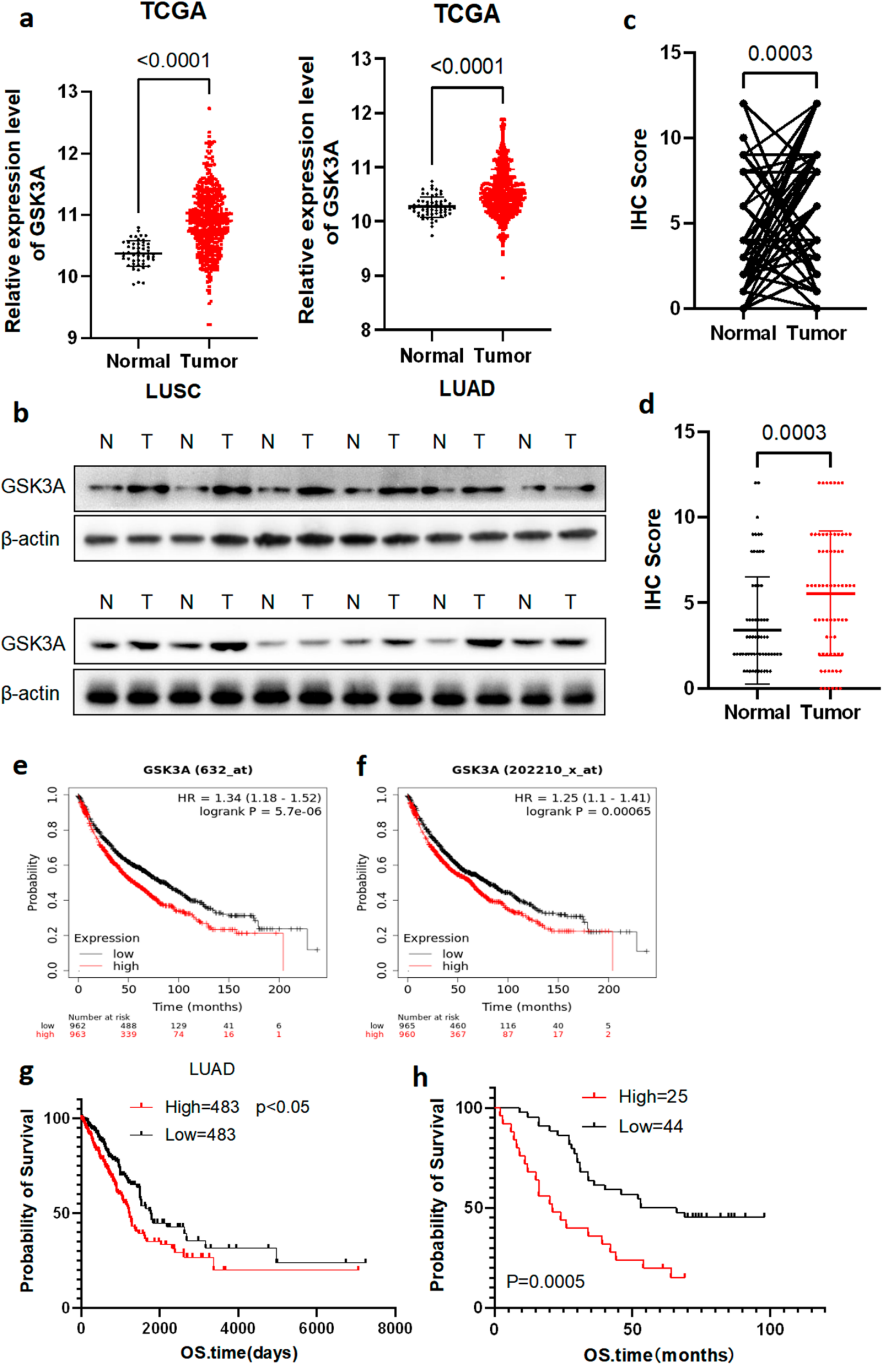
GSK3α was upregulated in NSCLC. **a** Profiles of GSK3α expression in NSCLC in a TCGA dataset; *p* < 0.0001. **b** Western blots showing levels of GSK3α protein in NSCLC tumor and matched normal tissues. **c, d** IHC results showing levels of GSK3α expression in NSCLC patients; *p* = 0.0003. **e, f** Kaplan–Meier curves showing profiles of overall survival times of patients grouped by GSK3α expression level; *p* < 0.05. **g** Overall survival times of patients grouped by GSK3α expression level based on a TCGA dataset; *p* < 0.05. **h** 70 patients were grouped by GSK3α expression level using IHC assays, and the overall survival time of patients were analyzed using Kaplan–Meier Plotter; *p* < 0.05

To determine levels of GSK3α expression in NSCLC, we searched the TGCA and GEPIA databases. Results showed that GSK3α was significantly upregulated in both lung adenocarcinoma, lung squamous carcinoma and various cancer, relative to adjacent normal tissues (Fig. 1a). Similarly, analysis of clinical NSCLC samples revealed that GSK3α was also upregulated (Fig. 1b–d and Additional file 2: Fig. S1b). Next, we generated Kaplan–Meier curves to investigate prognosis of patients overexpressing GSK3α using a TCGA dataset, alongside clinical samples and found that patients with higher levels of GSK3α expression exhibited worse overall survival times (Fig. 1e–h and Additional file 2: Fig. S1c). Consistently, data from Human Protein Atlas showed that liver cancer and endometrial cancer patients with higher levels of GSK3α expression exhibited worse overall survival times than their low-expression counterparts (Additional file 2: Fig. S1d–e). Collectively, these findings showed that GSK3α was upregulated in NSCLC, and this expression level was negatively correlated with overall survival times in patients.

### GSK3αoverexpression promoted proliferation, migration, invasion and colony formation ability of NSCLC cells

**Fig. 2:**
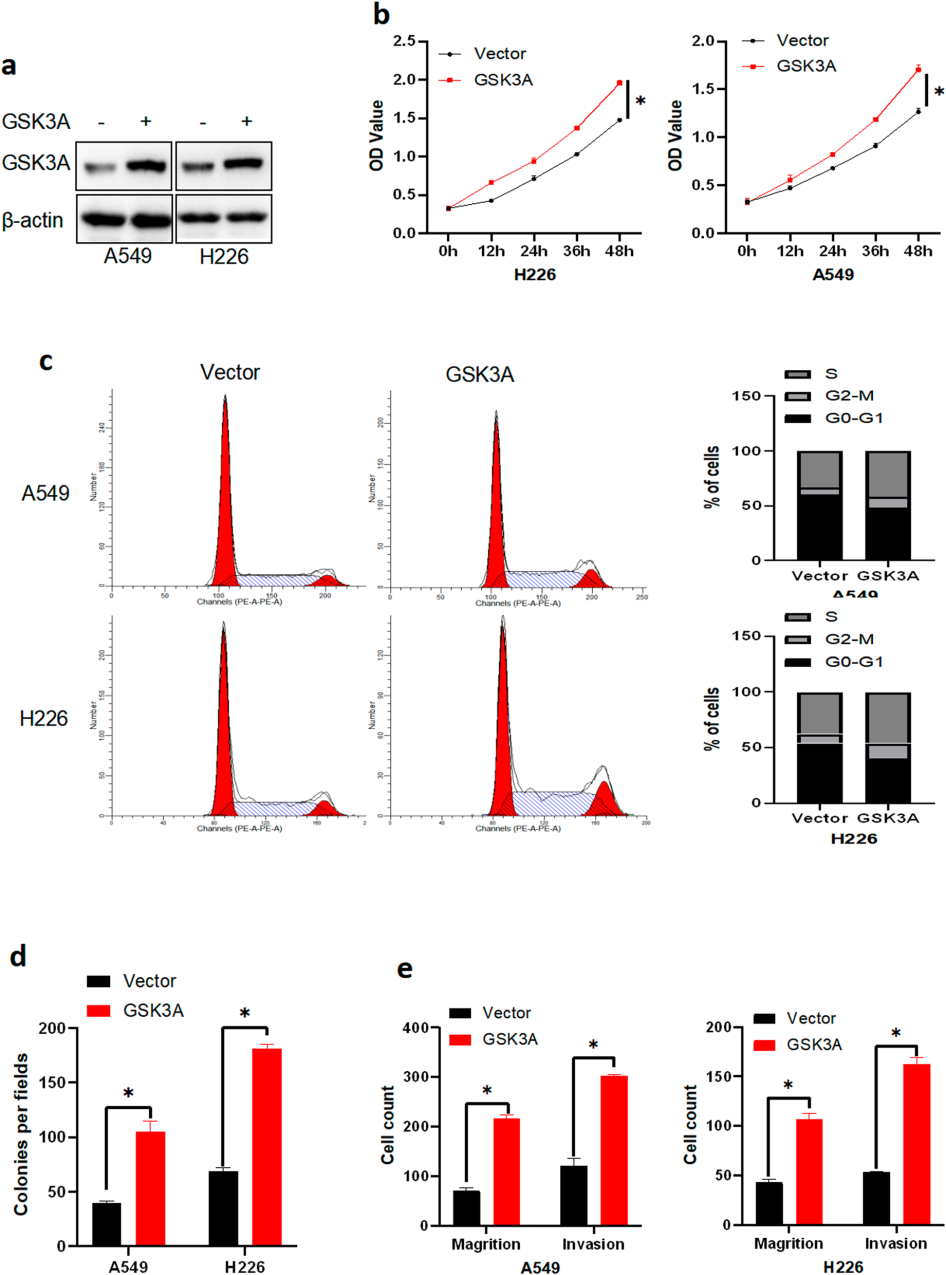
GSK3αoverexpression promoted tumorigenesis in NSCLC cells in vitro. **a** Western blots showing protein expression in cells transfected with vector or GSK3α lentivirus. **b** CCK8 assays results showing proliferation ability of NSCLC cells stably expressing vector or GSK3α; **p < 0.05. **c** PI assay results showing profiles of the cell cycle in NSCLC cells stably expressing vector or GSK3α. **d** Colony formation ability of NSCLC cells stably expressing vector or GSK3α; *p* < 0.05. **e** Transwell assays results showing migration and invasion ability of NSCLC cells stably expressing vector or GSK3α; **p < 0.05

To understand the biological function of GSK3α, we stably expressed GSK3α or empty vectors in NSCLC cells (A549 and H226) using lentivirus (Fig. 2a), then performed CCK8 and cell cycle assays to assess whether GSK3α could regulate cell proliferation and cell cycle progression. Results showed that overexpressing GSK3α promoted proliferation of NSCLC cells. Notably, cells with higher levels of GSK3α expression had a lower G0–G1 phase but a higher S and G2-M phase ratio (Fig. 2b–c). Moreover, results from colony formation assays showed that cells with higher levels of GSK3α expression had higher colony formation ability (Fig. 2d and Additional file 2: Fig. S2a).

Next, we hypothesized that GSK3α might affect cell migration and invasion ability, thus performed Transwell analysis to ascertain this. Interestingly, GSK3α overexpression mediated an increase in cell number on the lower membrane, indicative of higher migration and invasion abilities (Fig. 2e and Additional file 2: Fig. S2b). Taken together, these findings demonstrated that GSK3α upregulation promotes proliferation, migration, invasion and colony formation ability of NSCLCs.

### Loss of GSK3α inhibited proliferation, migration, invasion and colony formation ability of NSCLC cells

**Fig. 3:**
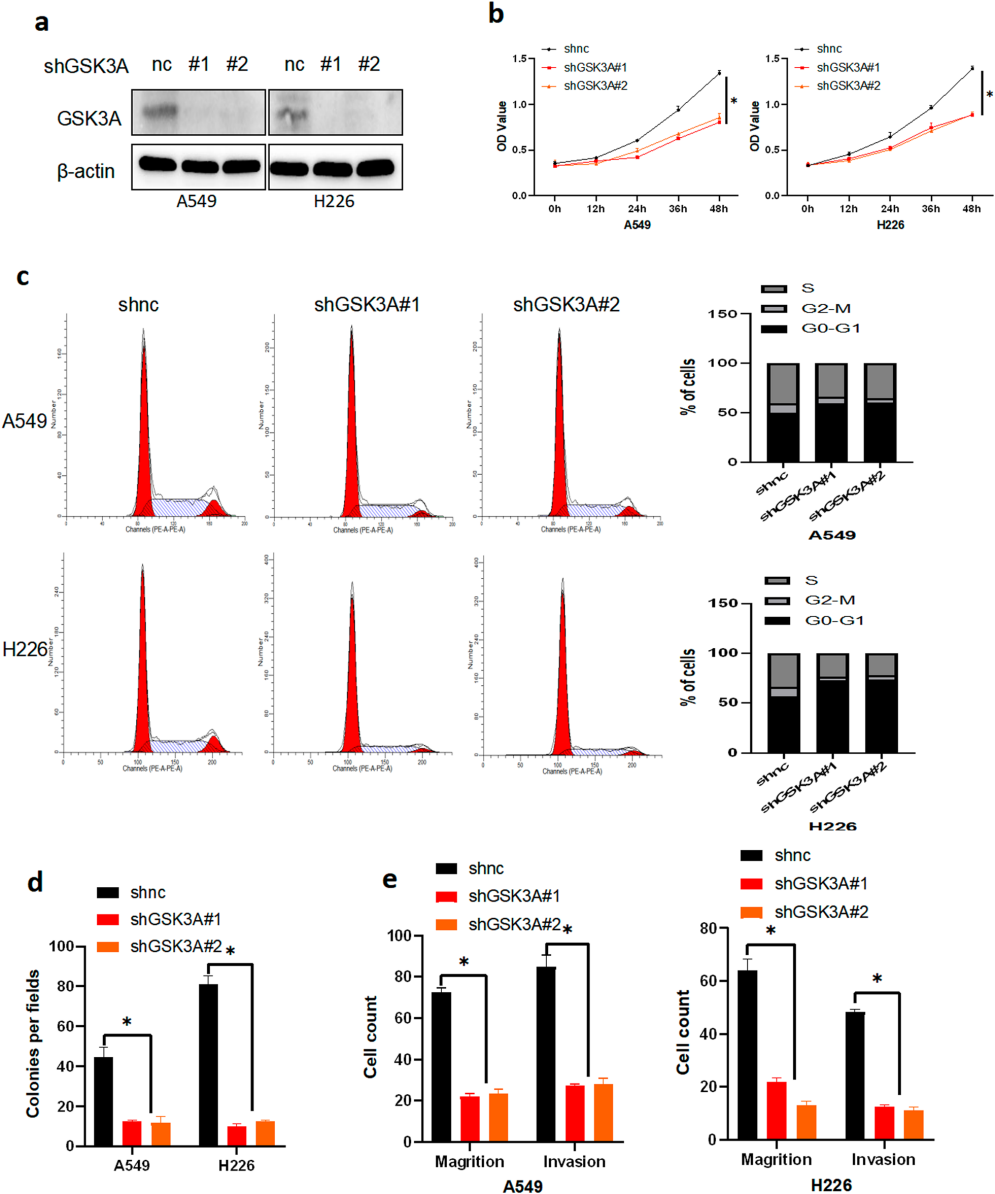
Loss of GSK3α inhibited proliferation, colony formation, migration and invasion ability in NSCLC cells. **a** Western blots showing successful transfection of cells with shnc or shGSK3α lentivirus. **b** CCK8 assays results showing proliferation ability of NSCLC cells stably expressing shnc or shGSK3α; **p < 0.05. **c** PI assay results showing the cell cycle profiles of NSCLC cells stably expressing shnc or shGSK3α. **d** Colony formation ability of NSCLC cells stably expressing shnc or shGSK3α; *p* < 0.05. **e** Transwell assay results showing migration and invasion ability of NSCLC cells stably expressing shnc or shGSK3α; **p < 0.05

To further validate GSK3α’s biological function, we used a lentivirus vector to silence GSK3α expression in A549 and H226 cell lines (Fig. 3a). Results from CCK8 and cell cycle analyses showed that loss of GSK3α inhibited proliferation ability of NSCLC cells, mediated an increase and a decrease in G0-G1 and G2-M phase ratios, respectively (Fig. 3b–c). Results from colony formation assays showed that silencing of GSK3α inhibited colony formation ability of NSCLCs (Fig. 3d and Additional file 2: Fig. S2c), while Transwell analysis results further revealed that loss of GSK3α inhibited migration, and invasion ability of NSCLC cells (Fig. 3e and Additional file 2: Fig. S2d). Overall, these results demonstrated that levels of GSK3α expression were positively correlated with proliferation, migration, invasion and colony formation ability of NSCLC cells.

### GSK3α plays a role in tumor angiogenesis

Previous studies have shown that during tumor progression, immature vessels are gradually formed, also in a process known as tumor angiogenesis [23]. During this process, cancer cells secrete pro-angiogenesis factors, namely VEGFA, and PDGFB, which accelerate their progression [26]. Accumulating research evidences have shown that immature vessel formation could subsequently promote tumor progression [27]. With the HIF1/VEGFA signaling pathway shown to play a crucial role in tumor angiogenesis [28].

**Fig. 4:**
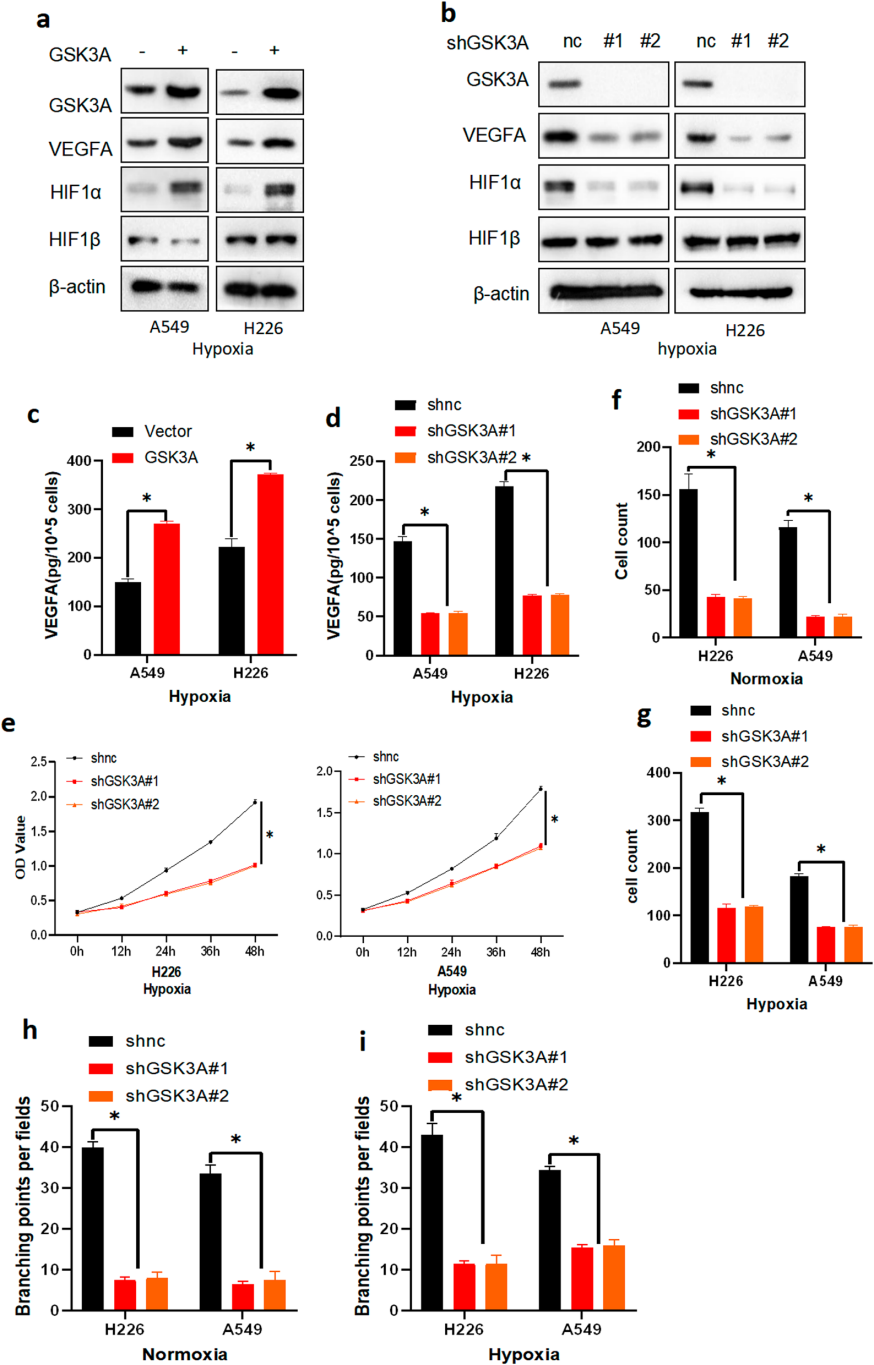
GSK3αactivated the HIF1/VEGFA signal pathway in NSCLC tumors. **a, b** Western blots showing levels of protein expression in cells under hypoxia. **c, d** ELISA results showing levels of VEGFA expression in conditional medium of identified cells under hypoxia; *p* < 0.05. **e** CCK8 assay results demonstrating proliferation ability of HUVECs cultured in different conditional media; *p* < 0.05. **f, g** Transwell assay results showing migration ability of identified HUVECs; *p* < 0.05. **h, i** Results of tube formation assays demonstrating tube formation ability of HUVECs cultured in conditional media; *p* < 0.05

In the present study, results from analysis of the GSE17475 dataset revealed marked activation of the hypoxia signaling pathway in patients with higher levels of GSK3α expression (Additional file 2: Fig. S3a). Consequently, we investigated whether GSK3α regulates tumor angiogenesis. Interestingly, we found that GSK3α overexpression upregulated HIF1α and VEGFA expression, but not HIF1β, in both normoxia and hypoxia (Fig. 4a and Additional file 2: Fig. S3b). Conversely, loss of GSK3α downregulated HIF1α and VEGFA expression, but not HIF1β, in both normoxia and hypoxia (Fig. 4b and Additional file 2: Fig. S3c). ELISA results indicated that levels of GSK3α expression were positively correlated with those of VEGFA in both normoxia and hypoxia (Fig. 4c–d and Additional file 2: Fig. S3d–e).

To further validate the novel role of GSK3α in tumor angiogenesis, we cultured endothelial cells (HUVECs) in conditional media (CM) from NSCLC cells expressing shnc or shGSK3α, then performed CCK8 assay. Results showed that HUVECs cultured in CM from shGSK3α cells had lower proliferation ability in both normoxia and hypoxia compare to CM from shnc cells (Fig. 4e and Additional file 2: Fig. S3f). Next, we performed Transwell assays to assess the migration ability of HUVECs cultured in different media, and found that HUVECs cultured in CM from shGSK3α cells had lower migration ability in both normoxia and hypoxia compare to CM from shnc cells (Fig. 4f–g and Additional file 2: Fig. S3g–h). Similarly, results from tube formation assays further revealed significantly lower tube formation numbers in HUVECs cultured in CM from NSCLC cells expressing shGSK3α in both normoxia and hypoxia, relative to those cultured with CM from NSCLC cells expressing shnc (Fig. 4h–i and Additional file 2: Fig. S3i–j). Collectively, these data strongly implied that GSK3α regulated tumor angiogenesis via the HIF1α/VEGFA signaling pathway.

### GSK3α independently regulated the HIF1α/VEGFA signaling pathway by modulating PHDs-pVHL-mediated HIF1α poly-ubiquitin

**Fig. 5:**
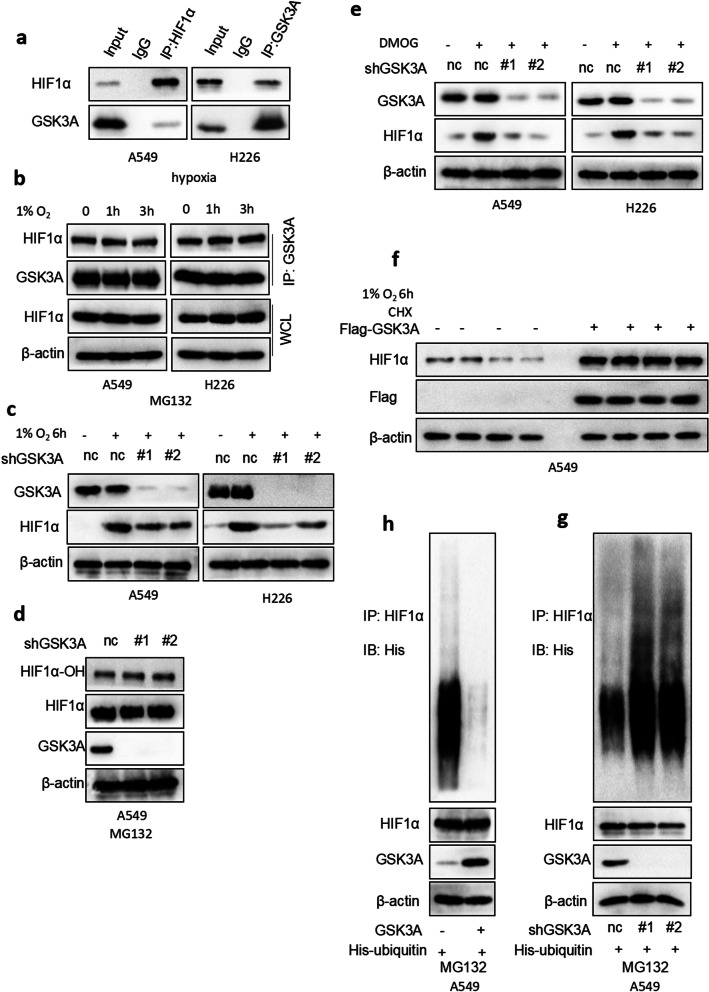
GSK3αregulates HIF1α stabilization in an PHDs-pVHL complex independent way. **a** Immunoprecipitation and Western blot results showing levels of protein expression in A549 and H226 cells. **b** Immunoprecipitation and Western blot results showing levels of proteins expression in A549 and H226 cells under hypoxia conditions. MG132 was used to inhibit endogenous protein degradation. **c, e** Western blots showing expression of proteins from identified cells under hypoxia or DMOG conditions. **d** Silencing GSK3A expression using lentivirus in A549 cells, MG132 was used to inhibit endogenous protein degradation, followed by Western blot analysis. **h** Immunoprecipitation and Western blot results showing protein expression in A549 cells transfected with His-ubiquitin plasmid and stably expressing Vector or GSK3α. MG132 was used to inhibit endogenous protein degradation. **g** Immunoprecipitation and Western blot results showing protein expression in A549 cells transfected with His-ubiquitin plasmid and stably expressing shnc or shGSK3α. MG132 was used to inhibit endogenous protein degradation

Previous studies have shown that levels of HIF1α expression are mainly modulated by both transcriptional and post-transcriptional regulation [29]. To further investigate the mechanism underlying activation of GSK3α-mediated HIF1α/VEGFA signaling pathway, we first used qRT-PCR to quantify levels of HIF1α mRNA in NSCLC cells expressing shnc or shGSK3α. Results revealed no statistically significant differences in HIF1α expression in NSCLC cells expressing shnc or shGSK3α in both normoxia and hypoxia (Additional file 2: Fig. S4a–b). Interestingly, results from Co-IP showed that HIF1α bound to GSK3α under hypoxia conditions (Fig. 5a). Next, we used MG132 to inhibit HIF1α degradation in normoxia, then performed Co-IP analysis. Results showed that GSK3α successfully bound to HIF1α under normoxia (Additional file 2: Fig. S4c). Moreover, we found that the interaction between HIF1α and GSK3α had no visual effect under hypoxia conditions (Fig. 5b). Taken together, results from Figs. 4a–b and 5b implied that GSK3α’s regulation of HIF1α expression levels was independent of its hydroxylation. To validate this hypothesis, we first subjected NSCLC cells expressing shnc or shGSK3α to hypoxia conditions, then performed western blot analysis to check protein expression. Results showed that silencing GSK3α effectively reversed hypoxia-induced HIF1α upregulation (Fig. 5c), but had no observable effect on levels of HIF1α hydroxylation (Fig. 5d). Furthermore, we used dimethyloxalylglycine (DMOG) [30], a prolyl hydroxylase inhibitor, to attenuate HIF-1α hydroxylation, and found that silencing of GSK3α could also downregulate HIF1α expression (Fig. 5e). Next, we used protein synthesis inhibitor (cycloheximide) to inhibit endogenous protein expression, and found that GSK3α overexpression markedly increased the half-life time of endogenous HIF-1a (Fig. 5f). Poly-ubiquitin-dependent protein degradation is the main mechanism underlying post-transcriptional regulation of HIF1α. Therefore, we investigated whether GSK3α regulates levels of HIF1α poly-ubiquitin. To this end, we transfected His-ubiquitin plasmid into A549 cells stably expressing Vector or GSK3α, then used MG132 to inhibit endogenous protein degradation. Interestingly, we found that GSK3α overexpression dramatically downregulated (Fig. 5g) while its silencing upregulated (Fig. 5h) poly-ubiquitin levels of HIF1α. Taken together, these findings showed that GSK3α independently regulated HIF1α poly-ubiquitin levels via the PHDs-pVHL complex.

### GSK3α’s regulation of tumor angiogenesis dependent on HIFα expression in vitro

**Fig. 6:**
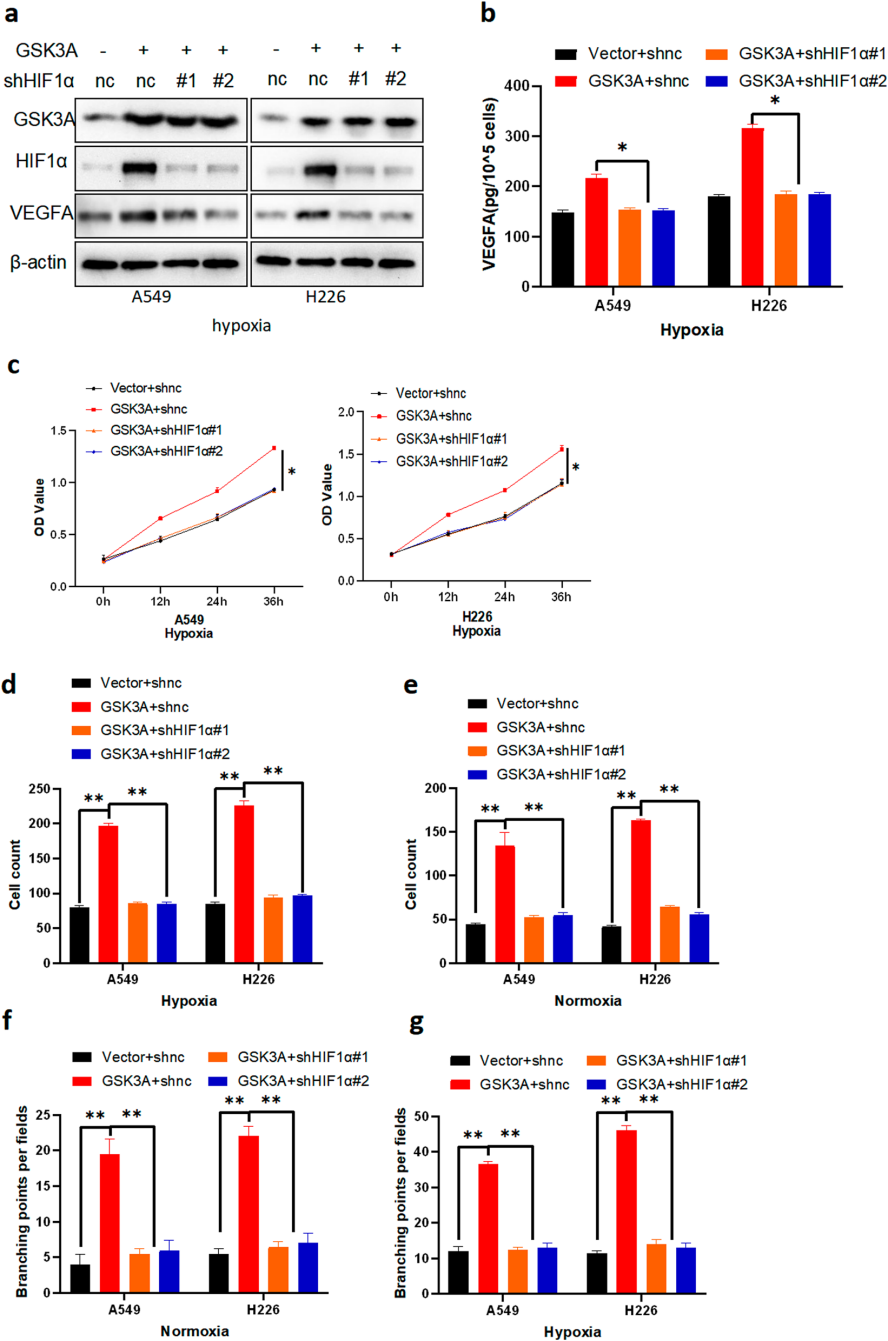
GSK3αregulated tumor angiogenesis in a HIF1α-dependent manner in vitro. **a, b** Construction of NSCLC cell lines stably expressing vector + shnc, GSK3α + shnc, GSK3α + shHIF1α#1, and GSK3α + shHIF1α#2 in hypoxia. Successful transfection was confirmed via Western blot (**a**), and ELISA (**b**) analyses; *p < 0.05. **c, d, g** HUVECs cultured in different conditional media under hypoxia, followed by transwell assays (**c**), CCK8 assays (**d**), and tube formation assays (**g**); *p < 0.05, **p < 0.05. **e, f** HUVECs cultured in different conditional media under normoxia, and analyzed by transwell assays (**e**), and tube formation assays (**f**); **p < 0.05

To further investigate the cross-talk between the GSK3α and HIF1/VEGFA signaling pathways, we stably expressed vector + shnc, GSK3α + shnc, GSK3α + shHIF1α#1, and GSK3α + shHIF1α#2 in NSCLC cell lines, then performed western blot and ELISA assays to analyze protein expression. Western blots showed that upregulation of GSK3α-related proteins could be reversed by silencing of HIF1α under both normoxia and hypoxia conditions (Fig. 6a and Additional file 2: Fig. S4d). ELISA results corroborated these findings (Fig. 6b and Additional file 2: Fig. S4e). Furthermore, we cultured HUVECs in conditional medium from NSCLC cell lines stably expressing vector + shnc, GSK3α + shnc, GSK3α + shHIF1α#1, and GSK3α + shHIF1α#2, then performed CCK8, Transwell, and tube formation assays. Results showed that HUVECs cultured in CM from cells stably expressing GSK3α exhibited significantly higher proliferation, migration, and tube formation abilities compared to those from cells stably expressing vector. Interestingly, silencing HIF1α effectively reversed these phenomena (Fig. 6c–g and Additional file 2: Fig. S4e–h). Taken together, these observations showed that GSK3α-mediated tumor angiogenesis depends on HIF1α expression.

### GSK3α regulates tumor angiogenesis in vivo, depending on HIF1α expression

**Fig. 7:**
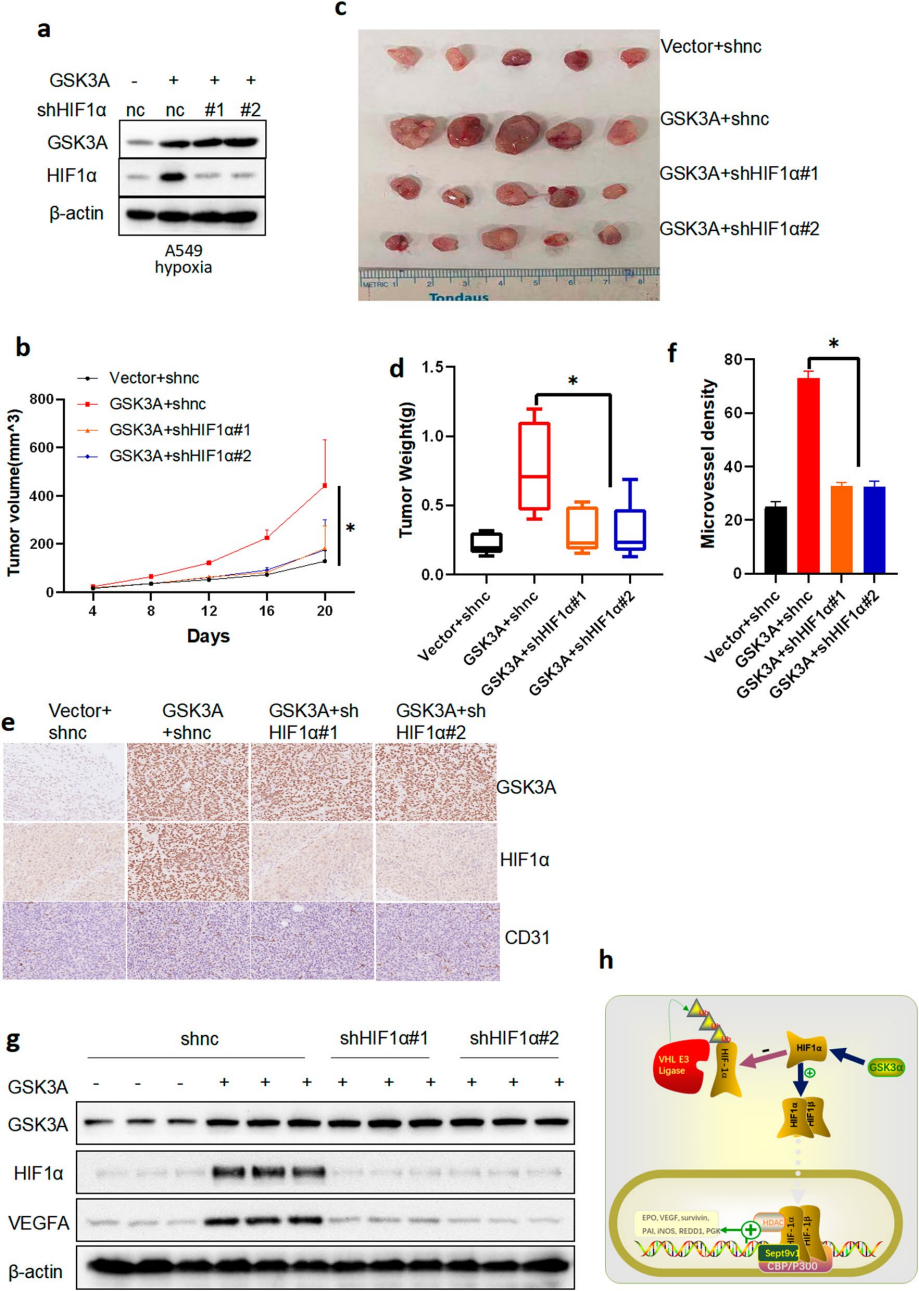
GSK3α regulated tumor angiogenesis in a HIF1α-dependent manner in vivo. **a** Construction of A549 cell lines stably expressing vector + shnc, GSK3α + shnc, GSK3α + shHIF1α#1, and GSK3α + shHIF1α#2. Successful transfections were confirmed via western blot analysis. **b** Tumor growth curve; *p < 0.05. **c** Tumor images. **d** Tumor weight; *p < 0.05. **e** IHC assay results showing the micro-vessel density of xenograft tumors. **f** Statistical analysis for the micro-vessel density of xenograft tumors; *p < 0.05. **g** Western blots showing protein expression in xenograft tumors. **h** The mechanism through which GSK3α regulates the HIF1/VEGFA signaling pathway

Next, we investigated whether GSK3α expression is correlated to that of HIF1α in vivo. To this end, we first stably expressed vector + shnc, GSK3α + shnc, GSK3α + shHIF1α#1, and GSK3α + shHIF1α#2 in A549 cells, then analyzed protein expression via western blot analysis (Fig. 7a). The four cell types were then subcutaneously injected into the abdomen of 4-week-old NOG nude mice. Analysis of tumor growth curve showed that cells expressing GSK3α + shnc grew faster than those expressing vector + shnc, while GSK3α promoting tumor growth could be inhibited by silencing of HIF1α (Fig. 7b). Results from analysis of tumor images and animal weights further confirmed that GSK3α promoting tumor growth could be inhibited by silencing of HIF1α (Fig. 7c and d). Next, we conducted IHC assays to investigate the micro-vessel density of tumors in the four groups and found that GSK3α markedly promoted tumor angiogenesis, although this could be inhibited by loss of HIF1α (Fig. 7e and f). Next, we investigated expression levels of GSK3α, HIF1α and VEGFA proteins in xenografted tumors via western blot assay and found that PRTM3 upregulated HIF1α and VEGFA expression. Besides, GSK3α promoting VEGFA expression could be inhibited by loss of HIF1α (Fig. 7g). Overall, these results demonstrated that GSK3α-inducing tumor angiogenesis was dependent on HIF1α.

## Discussion

GSK3α and GSK3β are members of the GSK3 family [10]. Although the role of GSK3β in tumorigenesis has been extensively studied, the biological function of GSK3αin this process remains largely unknown. Results of the present study demonstrated that GSK3α was significantly upregulated in lung cancer patients, who subsequently exhibited shorter survival times. Furthermore, we found that GSK3α overexpression could promote proliferation, migration, invasion and colony formation ability of cancer cells, while its loss resulted in an opposite phenomenon. These findings demonstrated that GSK3αfunctions as an oncogene in NSCLC tumorigenesis, which inspired us to explore detailed mechanism.

Although previous studies have suggested that angiogenesis could be an effective therapeutic strategy for treatment of cancer patients [31, 32]. The approach is commonly associated with drug-resistance [33]. Notably, research evidences have shown that patients with anti-angiogenesis resistance exhibit abnormal activation of the HIF1/VEGFA signaling pathway [24, 34]. However, the precise mechanism underlying this activation remains unknown, necessitating further explorations. The ubiquitin–proteasome way was the main manner to regulated HIF1α degradation. Under normoxia, HIF1α could be hydroxylated by PHDs, and then poly-ubiquitinated by VHL. However, HIF1α could also be degraded by an independent PHDs-pVHL signaling pathway [14]. Results of the present study showed that GSK3α regulates the HIF1/VEGFA signal pathway by independently stabilizing HIF1α via the PHDs-pVHL signaling pathway. Furthermore, our results showed that GSK3α overexpression could both stabilize and upregulate HIF1α expression. Interestingly, in our study, we found that GSK3β could not regulated HIF1α expression (Additional file 2: Fig. S3d). Recent studies showed that hypoxia could regulated GSK3β expression by HIF1α [35, 36]. However, our study showed that silencing of GSK3α could inhibit HIF1α expression, while had no effect on GSK3β expression. This discrepancy might be a reflection of the different biology exhibited by the different cell lines used in this study. In addition, the use of different experimental methods might contribute to this contrasting observation. GSK3α differed from GSK3β at its N- or C-terminal amino acid sequences. GSK3α and GSK3β had specific roles in cell survival, and GSK3α might paly a more important role in cancer cell survival and cancer treatment resistance [10]. Besides, GSK3β could shuttle from the nucleus to the cytoplasm, and GSK3β would be accumulated in the nucleus under proapoptotic stimuli [37]. In contrast, GSK3α had no ability to shuttle between the nucleus and the cytoplasm [10, 37]. GSK3α-mediated HIF1α expression was independent of its hydroxylation. Further validation of the cross-talk between GSK3α expression and regulation of the HIF1/VEGFA signaling pathway, both in vivo and in vitro, revealed that GSK3α-mediated cancer tumor angiogenesis was dependent on HIF1α expression in vitro. Results from in vivo assays proved that GSK3α-mediated tumorigenesis and tumor angiogenesis is dependent on levels of HIF1α expression.

## Conclusion

Overall, our results indicate that GSK3α functions as an oncogene in NSCLC, by regulating stabilization and expression of HIF1α in an independent manner via the PHDs-pVHL signaling pathway. Furthermore, we verified the cross-talk between GSK3α signal pathway and HIF1/VEGFA signal pathway. Taken together, these findings provide novel insights into the mechanism of GSK3α action, and are expected to guide future development of therapeutic strategies for cancer treatment.

## Supplementary Information

**Additional file 1. Table S1:** The primers of RT-PCR.

**Additional file 2: Figure S1.** GSK3α functions as an oncogene. **a**. Differential GSK3α expression between tumor and normal tissues across various types of tumors. **b, c**. Representative images of IHC assay results. **d, e**. Kaplan–Meier curves showing overall survival of patients, from the Human Protein Atlas database, grouped by GSK3α expression level. **Figure S2.** GSK3α acts as an oncogene. **a, c**. Representative images from colony formation assays. **b, d**. Representative images from the Transwell assays. **Figure S3.** GSK3α activated the HIF1/VEGFA signaling pathway. **a**. GSEA analysis results based on a GSE17475 dataset. **b, c.** Western blots showing levels of protein expression in identified cells under normoxia conditions. **d, e**. ELISA results showing levels of VEGFA expression in cells cultured in conditional medium under normoxia conditions; *p* < 0.05. **f**. CCK8 assay results showing proliferation ability of HUVECs cultured in different conditional media under normoxia conditions; *p* < 0.05. **g, h**. Representative images of Transwell assays performed on cells under hypoxia (**g**) or normoxia (**h**) conditions. **i, j**. Representative images of tube formation assay results performed in cells under hypoxia (**i**) or normoxia (**j**) conditions. **Figure S4.** GSK3α regulated tumor angiogenesis in a HIF1α-dependent manner. **a, b**. Levels of HIF1α mRNA expression in identified cells under hypoxia (**a**) or normoxia (**b**) conditions. **c**. Immunoprecipitation and Western blot results showing protein expression in A549 and H226 cells. MG132 was used to inhibit endogenous protein degradation. **d, e** Construction of NSCLC cell lines stably expressing vector + shnc, GSK3α + shnc, GSK3α + shHIF1α#1, and GSK3α + shHIF1α#2 in normoxia. Western blots (**d**), and ELISA results (**e**) were used to confirm successful transfection; *p < 0.05. **f**. CCK8 assay results in HUVECs cultured with different conditional media under normoxia conditions (**f**). **g, h**. Representative images of transwell assay results in cells under hypoxia (**g**) or normoxia (**h**) conditions. **i, j**. Representative images of tube formation assay results of cells under normoxia (**j**) or hypoxia (**i**) conditions.

## Data Availability

Not applicable.

## Abbreviations

GSK3α: The glycogen synthase kinase-3α
NSCLC: Non-small-cell lung cancer
PHDs: Prolyl hydroxylase domain family proteins
pVHL: The von Hippel–Lindau tumor suppressor protein
HIF1α: Hypoxia-inducible factor 1α
CM: Conditional medium
DMOG: Dimethyloxalylglycine

## References

[1] Bray F, Ferlay J, Soerjomataram I, Siegel RL, Torre LA, Jemal A (2018). Global cancer statistics 2018: GLOBOCAN estimates of incidence and mortality worldwide for 36 cancers in 185 countries. CA Cancer J Clin.

[2] Gridelli C, Rossi A, Carbone DP, Guarize J, Karachaliou N, Mok T (2015). Non-small-cell lung cancer. Nat Rev Dis Primers.

[3] Ettinger DS, Aisner DL, Wood DE, Akerley W, Bauman J, Chang JY (2018). NCCN guidelines insights: non-small cell lung cancer, version 5.2018. J Natl Compr Canc Netw.

[4] Chheang S, Brown K (2013). Lung cancer staging: clinical and radiologic perspectives. Semin Intervent Radiol.

[5] Torre LA, Bray F, Siegel RL, Ferlay J, Lortet-Tieulent J, Jemal A (2015). Global cancer statistics, 2012. CA Cancer J Clin.

[6] Hirsch FR, Scagliotti GV, Mulshine JL, Kwon R, Curran WJ, Wu Y-L (2017). Lung cancer: current therapies and new targeted treatments. The Lancet.

[7] Keith B, Simon MC (2007). Hypoxia-inducible factors, stem cells, and cancer. Cell.

[8] Brahimi-Horn MC, Chiche J, Pouyssegur J (2007). Hypoxia and cancer. J Mol Med (Berl).

[9] Jing X, Yang F, Shao C, Wei K, Xie M, Shen H (2019). Role of hypoxia in cancer therapy by regulating the tumor microenvironment. Mol Cancer.

[10] Darrington RS, Campa VM, Walker MM, Bengoa-Vergniory N, Gorrono-Etxebarria I, Uysal-Onganer P (2012). Distinct expression and activity of GSK-3alpha and GSK-3beta in prostate cancer. Int J Cancer.

[11] Zhou J, Freeman TA, Ahmad F, Shang X, Mangano E, Gao E (2013). GSK-3alpha is a central regulator of age-related pathologies in mice. J Clin Investig.

[12] Zhou J, Lal H, Chen X, Shang X, Song J, Li Y (2010). GSK-3alpha directly regulates beta-adrenergic signaling and the response of the heart to hemodynamic stress in mice. J Clin Investig.

[13] Macklin PS, McAuliffe J, Pugh CW, Yamamoto A (2017). Hypoxia and HIF pathway in cancer and the placenta. Placenta.

[14] Denko NC (2008). Hypoxia, HIF1 and glucose__metabolism in the solid tumour. Nat Rev Cancer.

[15] Giaccia A, Siim BG, Johnson RS (2003). HIF-1 as a target for drug development. Nat Rev Drug Discov.

[16] Semenza GL (2007). Hypoxia-Inducible Factor 1 (HIF-1) Pathway. Sci STKE.

[17] Sharp FR, Bernaudin M (2004). HIF1 and oxygen sensing in the brain. Nat Rev Neurosci.

[18] Kim Y, Nam HJ, Lee J, Park DY, Kim C, Yu YS (2016). Methylation-dependent regulation of HIF-1alpha stability restricts retinal and tumour angiogenesis. Nat Commun.

[19] Wang GL, Jiang BH, Rue EA, Semenza GL (1995). Hypoxia-inducible factor 1 is a basic-helix-loop-helix-PAS heterodimer regulated by cellular O2 tension. Proc Natl Acad Sci USA.

[20] Sano M, Minamino T, Toko H, Miyauchi H, Orimo M, Qin Y (2007). p53-induced inhibition of Hif-1 causes cardiac dysfunction during pressure overload. Nature.

[21] Viallard C, Larrivee B (2017). Tumor angiogenesis and vascular normalization: alternative therapeutic targets. Angiogenesis.

[22] Semenza GL (2003). Targeting HIF-1 for cancer therapy. Nat Rev Cancer.

[23] Tang CM, Yu J (2013). Hypoxia-inducible factor-1 as a therapeutic target in cancer. J gastroenterol hepatol.

[24] Chen C, Cai S, Wang G, Cao X, Yang X, Luo X (2013). c-Myc enhances colon cancer cell-mediated angiogenesis through the regulation of HIF-1alpha. Biochem Biophys Res Commun.

[25] Jain RK (2001). Normalizing tumor vasculature with anti-angiogenic therapy: a new paradigm for combination therapy. Nat Med.

[26] Harris AL (2002). Hypoxia—a key regulatory factor in tumour growth. Nat Rev Cancer.

[27] Semenza GL (2012). Hypoxia-inducible factors in physiology and medicine. Cell.

[28] Eswarappa SM, Fox PL (2015). Antiangiogenic VEGF-Ax: a new participant in tumor angiogenesis. Cancer Res.

[29] Lu X, Kang Y (2010). Hypoxia and hypoxia-inducible factors: master regulators of metastasis. Clin Cancer Res.

[30] Geng H, Liu Q, Xue C, David LL, Beer TM, Thomas GV (2012). HIF1alpha protein stability is increased by acetylation at lysine 709. J Biol Chem.

[31] Weidner N, Semple JP, Welch WR, Folkman J (1991). Tumor angiogenesis and metastasis–correlation in invasive breast carcinoma. N Engl J Med.

[32] Bartels K, Grenz A, Eltzschig HK (2013). Hypoxia and inflammation are two sides of the same coin. Proc Natl Acad Sci USA.

[33] Tarade D, Ohh M (2018). The HIF and other quandaries in VHL disease. Oncogene.

[34] Ramjiawan RR, Griffioen AW, Duda DG (2017). Anti-angiogenesis for cancer revisited: is there a role for combinations with immunotherapy?. Angiogenesis.

[35] Saieva L, Barreca MM, Zichittella C, Prado MG, Tripodi M, Alessandro R (2020). Hypoxia-induced miR-675-5p supports beta-catenin nuclear localization by regulating GSK3-beta activity in colorectal cancer cell lines. Int J Mol Sci.

[36] Mottet D, Dumont V, Deccache Y, Demazy C, Ninane N, Raes M (2003). Regulation of hypoxia-inducible factor-1alpha protein level during hypoxic conditions by the phosphatidylinositol 3-kinase/Akt/glycogen synthase kinase 3beta pathway in HepG2 cells. J Biol Chem.

[37] Meares GP, Jope RS (2007). Resolution of the nuclear localization mechanism of glycogen synthase kinase-3: functional effects in apoptosis. J Biol Chem.

