# Supplementary material for: Glycogen synthase kinase GSK3α promotes tumorigenesis by activating HIF1/VEGFA signaling pathway in NSCLC tumor
Source: Cell Commun Signal. 2022 Mar 15;20:32. doi: 10.1186/s12964-022-00825-3 (PMC8922767; doi:10.1186/s12964-022-00825-3)
Supplement: Supplementary file 2 — Additional file 1. Table S1: The primers of RT-PCR. [file 12964_2022_825_MOESM2_ESM.docx]

| Supplementary Table 1: the primers of RT-PCR | | |
| --- | --- | --- |
| Gene name | Forward: | Backward: |
| HIF1A | 5’-GAACGTCGAAAAGAAAAGTCTCG-3’ | 5’-CCTTATCAAGATGCGAACTCACA-3’ |
|  |  |  |
|  |  |  |
|  |  |  |
|  |  |  |
