# Supplementary material for: Glycogen synthase kinase GSK3α promotes tumorigenesis by activating HIF1/VEGFA signaling pathway in NSCLC tumor
Source: Cell Commun Signal. 2022 Mar 15;20:32. doi: 10.1186/s12964-022-00825-3 (PMC8922767; doi:10.1186/s12964-022-00825-3)

Supplementary Figure 1

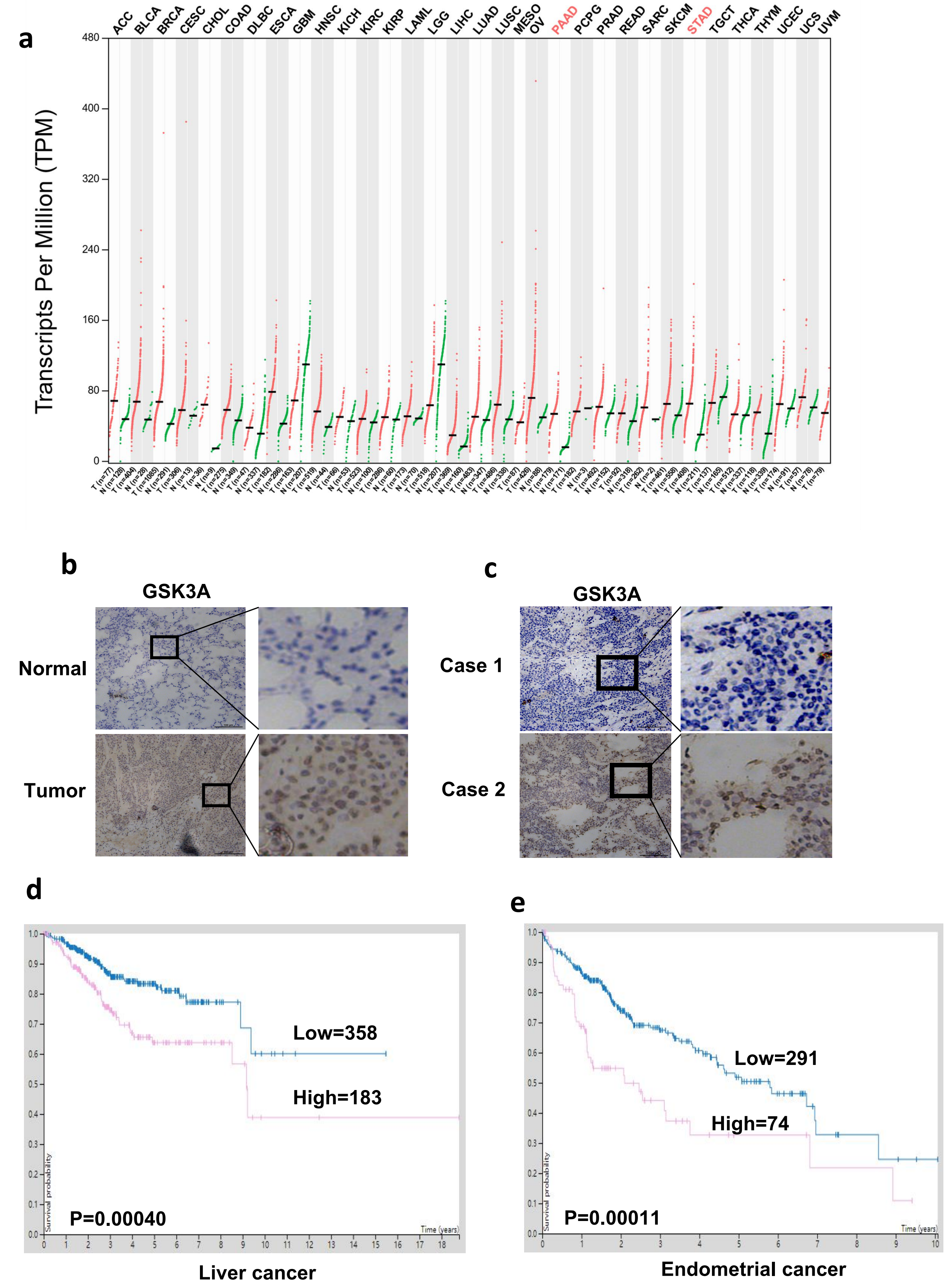

Supplementary Figure 2

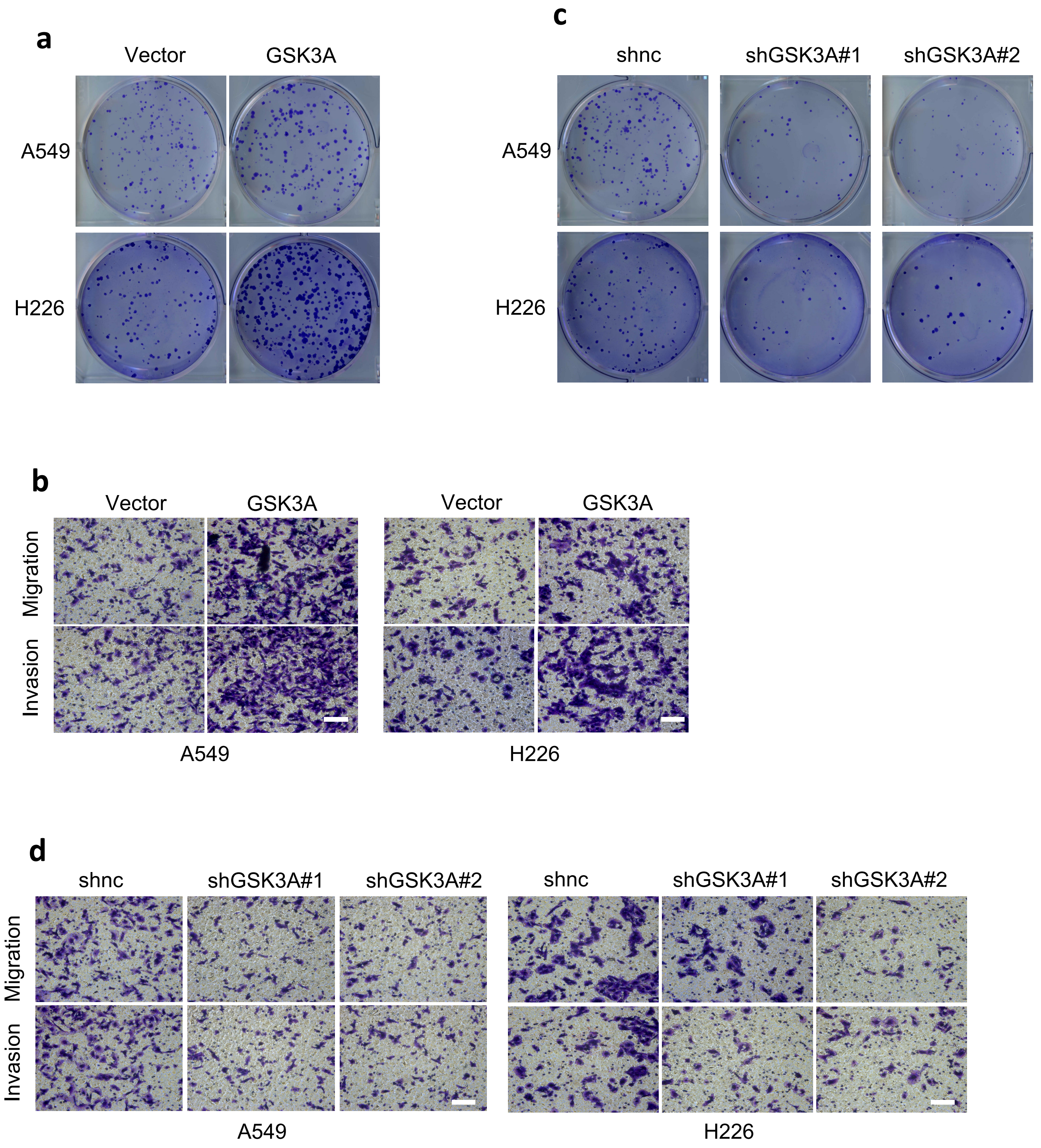

Supplementary Figure 3

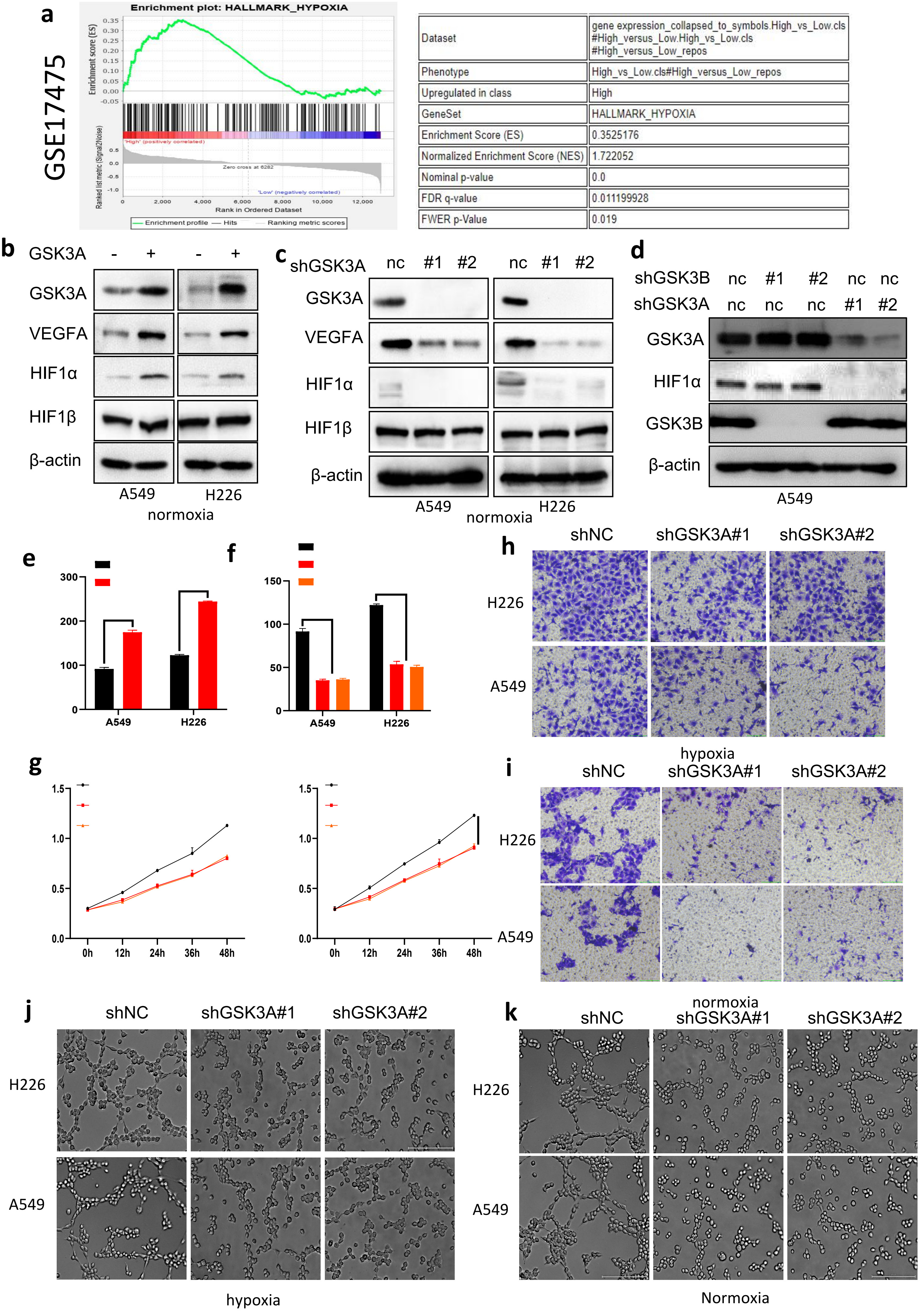

Dataset	gene expression_collapsed_to_symbols.High_vs_Low.cls #High_versus_Low.High_vs_Low.cls #High_versus_Low_repos
Phenotype	High_vs_Low.cls#High_versus_Low_repos
Upregulated in class	High
GeneSet	HALLMARK_HYPOXIA
Enrichment Score (ES)	0.3525176
Normalized Enrichment Score (NES)	1.722052
Nominal p-value	0.0
FDR q-value	0.011199928
FWER p-Value	0.019

Supplementary Figure 4

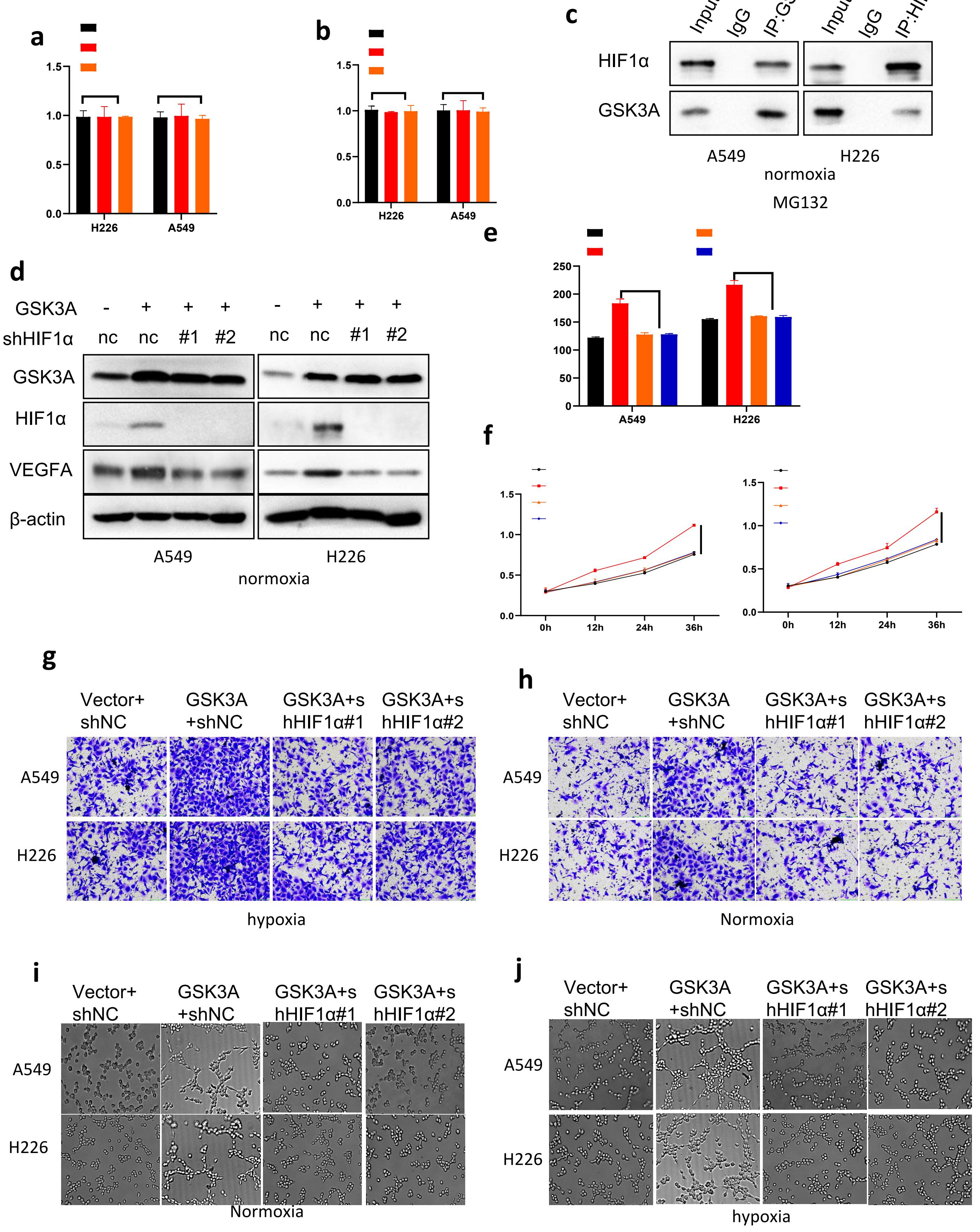

Supplement: Supplementary file 3 — Additional file 2: Figure S1. GSK3α functions as an oncogene. a. Differential GSK3α expression between tumor and normal tissues across various types of tumors. b, c. Representative images of IHC assay results. d, e. Kaplan–Meier curves showing overall survival of patients, from the Human Protein Atlas database, grouped by GSK3α expression level. Figure S2. GSK3α acts as an oncogene. a, c. Representative images from colony formation assays. b, d. Representative images from the Transwell assays. Figure S3. GSK3α activated the HIF1/VEGFA signaling pathway. a. GSEA analysis results based on a GSE17475 dataset. b, c. Western blots showing levels of protein expression in identified cells under normoxia conditions. d, e. ELISA results showing levels of VEGFA expression in cells cultured in conditional medium under normoxia conditions; p < 0.05. f. CCK8 assay results showing proliferation ability of HUVECs cultured in different conditional media under normoxia conditions; p < 0.05. g, h. Representative images of Transwell assays performed on cells under hypoxia (g) or normoxia (h) conditions. i, j. Representative images of tube formation assay results performed in cells under hypoxia (i) or normoxia (j) conditions. Figure S4. GSK3α regulated tumor angiogenesis in a HIF1α-dependent manner. a, b. Levels of HIF1α mRNA expression in identified cells under hypoxia (a) or normoxia (b) conditions. c. Immunoprecipitation and Western blot results showing protein expression in A549 and H226 cells. MG132 was used to inhibit endogenous protein degradation. d, e Construction of NSCLC cell lines stably expressing vector + shnc, GSK3α + shnc, GSK3α + shHIF1α#1, and GSK3α + shHIF1α#2 in normoxia. Western blots (d), and ELISA results (e) were used to confirm successful transfection; *p < 0.05. f. CCK8 assay results in HUVECs cultured with different conditional media under normoxia conditions (f). g, h. Representative images of transwell assay results in cells under hypoxia (g) or normoxia ( [file 12964_2022_825_MOESM3_ESM.pdf]
